# School-to-work transition: The role of life satisfaction, risk perception, and resilience in youth career decision-making

**DOI:** 10.1371/journal.pone.0339485

**Published:** 2025-12-23

**Authors:** Petar Stanimirović, Tea Borozan, Katarina Petrović, Dragan Bjelica, Zorica Mitrović, Marko Mihić, Dejan Petrović, Anđelija Đorđević Tomić

**Affiliations:** Department for Management and Project Management, Faculty of Organizational Sciences, University of Belgrade, Belgrade, Serbia; University College London, UNITED KINGDOM OF GREAT BRITAIN AND NORTHERN IRELAND

## Abstract

Young people often face uncertainty during the transition from education to work, along with high unemployment and job dissatisfaction, which is addressed in the EU Youth Strategy, highlighting the need for better career support. This study aimed to identify main factors influencing youth career decisions and to develop a decision-making model. Five core constructs were defined through literature review: Dealing with Uncertainty, Risk Preference, Adaptability and Resilience, Education and Support, and Life Satisfaction. Data were collected from 673 engineering students. Regression analysis was used to test the proposed model and hypotheses, while Mann-Whitney and Kruskal-Wallis tests examined group differences. The developed model accounts for 46.2% (R² = 0.462) of the variability in students’ career choices. Adaptability and resilience emerged as the most influential factor (β = 0.557). Certain differences, for specific constructs, were also observed in relation to different groups of family income, gender and extracurricular activity engagement. The model supports more informed career decisions and provides insights that may help improve career guidance and educational policy. The findings also may contribute to bridging theory and practice in career development research. The study is limited by its sample, which included only engineering students from the Republic of Serbia, potentially restricting the generalizability of the results.

## Introduction

Among all the life decisions individuals face, career choices stand out as one of the most important decisions [1]. The rapid growth of the ICT sector in the 21st century has intensified the frequency of these decisions due to abundant options and varying-quality information, intensifying the employment challenges and job-seeking pressures [2]. Career decision-making (CDM) involves choosing an educational path, entering the job market, staying or leaving a position, switching careers, or pursuing further training. These choices are often irreversible and carry lasting consequences [3].

Higher education plays a crucial role in talent cultivation, yet societal recognition of students as professionals, remains low, making career decisions more challenging [4]. College students undergo significant identity transformation as they transition from academia to professional life, often struggling with poor CDM, which negatively affects their social, personal, and professional well-being [5,6]. For young people who lack a clearly defined or long-held career aspiration, making a career decision can be a challenging and often overwhelming process [7].

The European Union Youth Strategy 2019–2027 recognizes labour market challenges as one of key concern [8], while a Deloitte study emphasized the need for alignment of educational programs and professional development opportunities with labour market trends [9]. The transition remains challenging, as around four in ten young individuals (25–29) have not yet obtained secure employment and are three times more likely to be unemployed compared to adults (aged 30 and above), with one in every two young people expressing dissatisfaction with their job [10]. These data clearly demonstrate that the challenges young people face in career decision-making are substantial, underlining the need to investigate and understand the underlying factors that shape this process.

Current research on CDM lacks sufficient empirical research to understand CDM processes and enhance the quality of CDM among college students [4]. Scholars like Phillips and Pazienza [11] emphasize that evaluating the processes leading to decisions is a crucial approach to assessing their quality. Building on this approach, enhancing university students’ CDM skills requires a thorough examination of the key factors influencing their career choices [2,3]. Afterwards, adopting CDM models can help individuals address challenges such as lack of readiness, insufficient information, limited decision-making knowledge, anxiety about choice or commitment, internal or external conflicts, and lack of resources [12].

Therefore, the primary objective of this study is to identify factors influencing CDM, understand CDM processes, and develop a model that not only assists university students in making informed decisions but also provides practical guidance to organizations and policymakers working with youth. By conducting a detailed literature review, key factors influencing the CDM process will be identified, laying the foundation for the proposed model and questionnaire structure. The analysis of the collected data will determine the significance of these factors, providing meaningful insights and guiding the formulation of relevant recommendations.

## Theory and hypotheses

Theoretical approaches to career development provide important guidance for explaining how young people make career decisions. Social Cognitive Career Theory (SCCT) emphasizes the role of self-efficacy, outcome expectations, and personal goals in shaping interests and career paths [13]. Career Construction Theory (CCT) highlights the process of career identity development, where adaptability and proactive agency are essential resources for navigating changing life and work contexts [14]. The Theory of Work Adjustment (TWA) focuses on the correspondence between individuals and their work environments, suggesting that satisfaction and stability arise when personal needs and abilities align with workplace requirements [15]. Although distinct, these frameworks converge on two key points: career decision-making reflects the dynamic interaction of individual traits and contextual conditions, and effective career choices require both adaptability and supportive structures.

Following these theories and cross-referencing them with other scientific literature, several factors are expected to explain variation in how young people approach career decisions. Youth-specific attitudes and personality traits shed additional light on career processes [16,17]. Perceptions of risk are multifaceted, while some view it as a threat to stability, others argue that risk-taking is essential for success, personal growth, and career development, provided it is undertaken with caution [18,19]. Similarly, flexibility and adaptability are crucial. Some young people remain open to pursuing better job opportunities as they arise, while others actively seek positions aligned with their future goals and lifestyle [17]. These orientations illustrate how proactive engagement interacts with structural opportunities to shape career trajectories.

**Fig 1:**
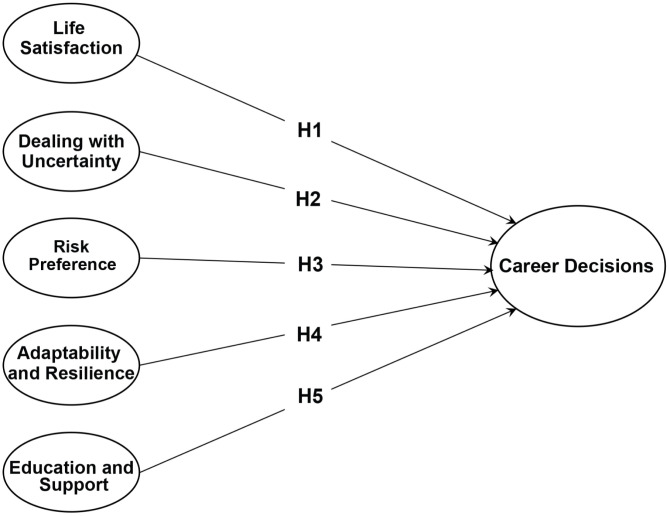
Hypothesized model.

Taken together, these arguments suggest that career decision-making among youth results from the combined effects of psychological background and attitudes toward risk and uncertainty. On this basis, the study formulates a set of hypotheses that define expected relationships between the identified factors and career decision-making. The hypotheses are presented in the following sections, and a conceptual model in Fig 1 summarizes the proposed framework tested in this study:

### Youth life satisfaction and career choices

Life satisfaction (LS) is generally understood as an overall evaluation of one’s quality of life [20], which often includes the extent to which personal goals have been achieved [21]. While personality traits such as optimism can increase LS, researchers highlight that enduring satisfaction is more strongly shaped by accumulated experiences and external circumstances [22].

For young people, the transition from education to the workforce is a critical stage that directly affects how satisfied they feel with life [23]. During this period, their well-being is particularly sensitive to socio-economic conditions, such as financial stability, as well as to the presence of family and peer support [24]. Structural conditions, such as access to healthcare, housing, and quality public services, also make a substantial contribution to life satisfaction [25]. At the same time, financial security matters as well. Young people who perceive their financial situation positively report higher LS [26]. Yet, evidence shows that pursuing higher income without meaningful employment can reduce well-being, often by raising stress and cutting leisure time [27].

Health is another essential component of life satisfaction. Poor health generally lowers LS, while good health, intrinsic motivation, a positive physical self-concept, and regular physical activity significantly enhance it [28,29]. Beyond present conditions, having a positive outlook on the future and strong planning skills further strengthen LS, particularly among students who are in the process of making career-related choices [30,31].

Taken together, these findings suggest that higher life satisfaction provides young people with the resources, resilience, and outlook necessary to make more confident and adaptive career decisions. Based on this, the following hypothesis is proposed:

*Hypothesis 1: Higher life satisfaction positively affects career decision-making processes*

### Youth career decision-making under uncertainty

Uncertainty is an unavoidable part of modern career development, especially for young people transitioning from education to the labour market. Situations where outcomes cannot be predicted often shape how individuals evaluate their options and make decisions [32]. In such contexts, uncertainty does not stem only from external forces like economic instability or social change, but also from personal responses and coping strategies [33,34].

Young people often deal with uncertainty by reevaluating their plans and adjusting them in light of new information or changing circumstances. While this flexibility can be a resource, many students also report feeling stressed when facing uncertainty about their future [35]. To reduce anxiety, they frequently seek advice from others, especially mentors and family, showing that social support remains a key resource in career decision-making [36]. At the same time, a more cautious strategy involves delaying important life decisions until greater certainty is available. In contrast, some youth respond with avoidance, pushing decisions aside until they feel more secure [37].

Taken together, these findings suggest that uncertainty management in career decision-making involves both internal strategies (e.g., flexibility, coping with stress, reliance on advice) and external considerations (e.g., economic conditions, financial security). A balanced ability to recognize risks, regulate emotional responses, and mobilize resources may therefore enhance young people’s confidence and effectiveness in navigating career-related uncertainty. Based on these arguments, the following hypothesis is proposed:

*Hypothesis 2: The ability to cope with uncertainty has a significant positive effect on career decision-making.*

### Risk preference in youth career decision-making

Risk preferences represent a central factor in how young people approach career decision-making. In general terms, risk can be understood as the evaluation of potential outcomes that involve both possible gains and losses [38]. Previous research highlights that individuals differ in the degree to which they are willing to accept such risk levels: while some adopt risk-averse attitudes, others demonstrate risk-seeking behaviours that lead them to pursue new opportunities despite the presence of potential threats [39].

For many young people, risk aversion translates into delaying decisions until it becomes absolutely necessary. This pattern reflects an underlying belief that waiting reduces the chance of negative consequences, even if it also reduces opportunities for growth. Similarly, risk-averse youth often avoid seeking new opportunities altogether because they associate novelty with danger rather than potential reward [39]. A strong preference for stability is another common expression of risk aversion. Many students choose to remain in their comfort zone or prioritize stable and predictable job options over innovative or risky career paths. Avoiding career changes for fear of the unknown reflects the same protective orientation, where security and predictability outweigh potential benefits [40].

At the same time, risk preference does not always manifest as active decision-making. Some young people adopt a passive strategy, preferring to wait for opportunities to arise on their own rather than actively seeking them. While this approach reduces exposure to risk, it often leaves individuals dependent on external circumstances rather than proactive career management [12,41].

Overall, risk preferences shape the strategies youth adopt when facing career decisions. A stronger orientation toward risk avoidance may protect against immediate negative outcomes but can hinder exploration, innovation, and flexibility which are key skills in contemporary labour markets [42]. Conversely, a willingness to take risks and opportunities can open new career pathways but requires supportive structures and resources. Based on these considerations, the following hypothesis is proposed:

*Hypothesis 3: Greater risk preference has a significant positive effect on career decision-making.*

### Adaptability and resilience in youth career decision-making

Adaptability and resilience are increasingly recognized as essential resources for effective career decision-making. Adaptability refers to the ability to flexibly respond to changes, while resilience describes the capacity to recover from failure and continue progressing despite setbacks [43]. Together, these traits help young people navigate the complexity of modern labour markets where agile methodologies are widely used. Those who can adjust their plans in response to unexpected circumstances are better equipped to handle risks and demonstrate greater persistence in pursuing long-term goals [44,45].

Beyond formal schooling, additional training, such as developing digital competencies or retraining in technical fields, plays a pivotal role in building these qualities. Informal education strengthens students’ capacity to recognize opportunities and adapt to shifting career demands [40]. Furthermore, social networks are another important source of resilience. Support from family and friends provides emotional encouragement and practical advice, which can buffer against stress during career transitions [34,46]. Broader professional networks, including mentors and colleagues, further expand young people’s understanding of labour market requirements and increase their ability to adapt to unexpected challenges [36].

Taken together, these findings suggest that adaptability and resilience enable youth not only to withstand career-related setbacks but also to proactively seize new opportunities. Students with higher adaptability and resilience are more likely to make confident and informed career decisions. Based on these insights, the following hypothesis is proposed:

*Hypothesis 4: Higher levels of adaptability and resilience positively contribute to career decision-making.*

### Importance of education and support for youth career decision-making

Education has long been recognized as one of the strongest predictors of career outcomes. Higher education in particular is often perceived as a prerequisite for long-term stability and professional growth. Young people frequently associate the completion of higher or technical education with improved job opportunities and greater financial independence [47,48].

Empirical studies confirm this perception, showing that formal education accelerates skill acquisition, strengthens competitiveness, and facilitates successful entry into the labour market [48]. From this perspective, obtaining a diploma is not only an individual achievement but also a strategic investment in employability and stability [49]. At the same time, support from family and close social networks plays an equally important role in shaping career decision-making [36,46]. In this way, social support operates as a buffer against stress and a catalyst for proactive career development.

The importance of education and social support thus converges: while education provides the skills and qualifications necessary for labour market integration, support networks ensure the emotional and practical resources needed to use these opportunities effectively [41]. These arguments form the basis for the following hypothesis:

*Hypothesis 5: Education and Support significantly affect career decisions.*

## Research methodology

### Participants and procedure

**Fig 2:**
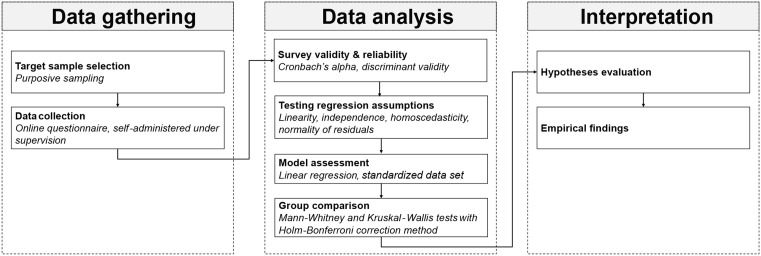
Research methodology.

The study was carried out as part of the project “*Engagement in Academic Achievements and Extracurricular Activities as Predictors of Life Satisfaction among High-school and University Students – SHINE”,* financed by the Science Fund of the Republic of Serbia, which primarily focused on life satisfaction and well-being among young individuals. This research paper specifically examines one aspect of the project, which was devoted to identifying factors influencing young people’s career choice and career decision-making. The study was conducted in the Republic of Serbia, where a total of 249,768 students are enrolled at universities, including 185,063 students pursuing first-degree studies, 64,656 students pursuing second- and third-degree studies and 49 students enrolled in older programs [50]. The primary focus of the study were undergraduate engineering students from the Republic of Serbia. The rationale for selecting this specific group of students was that, at this stage of their education, they encounter their first career decisions, such as choosing internships and first jobs. Since their field is in high demand, they face different career opportunities from the early stages of higher education, therefore such decisions can significantly influence their future career development. Fig 2 provides an overview of the research procedure, from data collection to final analysis.

Data collection was conducted from 21^st^ October to 24^th^ November 2024. The selected participants were informed about the questionnaire and completed it during class under supervision of a faculty staff member with no evaluative or grading authority over the participants, providing higher completion rates and resolving any issues [51], therefore ensuring increased reliability of the collected data. Prior to the survey, students were verbally informed about the study. Additionally, participants were presented with an introductory statement at the beginning of the questionnaire, which explained the study’s aims, the voluntary nature of participation, and the anonymous handling of their responses. By proceeding to complete the questionnaire, participants indicated their informed consent. Given the anonymous nature of the survey, written consent was not obtained to preserve confidentiality. This consent procedure, including the rationale for not collecting signed consent, was reviewed and approved by the Ethics committee of the Faculty of Organizational Sciences, University of Belgrade.

To collect data and evaluate the validity of the developed model, an online questionnaire incorporating the identified constructs was designed. The invitation to participate in the study was sent to 1,062 student email addresses. A total of 673 respondents completed the questionnaire, representing 63.37% of the sample. The research sample demonstrates a relatively balanced gender distribution, as shown in Table 1, which also includes additional sample characteristics. Fig 2 provides an overview of the procedure, from data collection to final analysis.

**Table 1:** Descriptive characteristics of the research sample.

Characteristics of respondents	Frequency (n)	Percent (%)	Characteristics of respondents	Frequency (n)	Percent (%)
**Gender**			**Family income**		
Male	278	41.3	500–700 €	66	9.8
Female	395	58.7	700–1,000 €	91	13.5
**Age**			1,000–2,500 €	242	36.0
18-20	594	88.3	2,500–5,000 €	229	34.0
21-23	71	10.5	>5,000 €	45	6.7
24-27	8	1.2	**Extracurricular activities engagement**		
**BMI category**			Every day	160	23.8
<18.5	50	7.4	Several times a week	340	50.5
18.5-25	503	74.7	Once a week	83	12.3
>25	120	17.8	Rarely or never	90	13.3
**Grade point average (GPA)**			**General health**		
5.00-6.50	118	17.5	Great	32	4.8
6.50-8.50	310	46.1	Good	39	58.8
8.50-10.00	245	36.4	Bad	245	36.4

### Measures

The initial version of the questionnaire was developed based on a review of the literature. It consisted of seven segments with a total of 71 items. Using Exploratory Factor Analysis (EFA), certain items were eliminated and grouped. The final version of the questionnaire comprised six segments and 48 items. Career Decisions (9 items), Dealing with Uncertainty (10 items), Perception of Risks & Opportunities (4 items), Adaptability & Resilience (9 items), and Life Decisions (6 items) were measured using a five-point Likert scale (1 = Strongly Disagree to 5 = Strongly Agree). Life Satisfaction (10 items) was assessed with the Brief Multidimensional Life Satisfaction Scale (BMLSS), measured on a seven-point scale ranging from 0 = Very dissatisfied to 6 = Very satisfied. The following Table 2 presents the defined constructs, their corresponding items, references, means and standard deviations.

**Table 2:** Constructs Overview.

Construct	Items	M ± SD	References
**Career Decisions**	*I expect to achieve financial independence before I turn 35.*	4.15 ± 0.825	[13,14–19]
	*I believe that taking risks is very important for achieving success in life.*	4.31 ± 0.799	
	*I believe that taking risks is sometimes necessary, but it should be done cautiously.*	4.16 ± 0.858	
	*I believe that taking risks is essential for personal growth through learning from experience.*	4.05 ± 0.844	
	*I am willing to take calculated risks to achieve my career goals.*	4.11 ± 0.881	
	*I believe that taking risks is necessary for career development and success.*	3.77 ± 0.944	
	*I am flexible when it comes to finding job opportunities.*	4.08 ± 0.881	
	*I am ready to pursue better job opportunities whenever they arise.*	3.78 ± 0.987	
	*I actively seek opportunities that align with my desired future career goals or lifestyle.*	5.924 ± 1.3347	
**Life Satisfaction**	*I would describe my satisfaction with my family life as…*	5.924 ± 1.3347	[20,21–31]
	*I would describe my satisfaction with my friendships as…*	5.990 ± 1.2143	
	*I would describe my satisfaction with my school/work situation as…*	5.525 ± 1.325	
	*I would describe my satisfaction with myself as…*	5.462 ± 1.3569	
	*I would describe my satisfaction with where I live as…*	5.562 ± 1.4214	
	*I would describe my overall life satisfaction as…*	5.743 ± 1.1729	
	*I would describe my satisfaction with my financial situation as…*	5.049 ± 1.4827	
	*I would describe my satisfaction with my future prospects as…*	5.776 ± 1.2095	
	*I would describe my satisfaction with my health as…*	5.744 ± 1.2910	
	*I would describe my satisfaction with my ability to handle daily life tasks as…*	5.574 ± 1.2141	
	*I would describe my satisfaction with my vitality/physical condition as…*	5.089 ± 1.5308	
**Dealing with Uncertainty**	*I often reevaluate my plans in order to adjust them to new information or circumstances.*	3.95 ± 0.874	[33,34–37]
	*I remain calm and focused when facing uncertainty about my future plans.*	3.53 ± 1.081	
	*I seek advice from others to better manage uncertainty in my career or personal life.*	3.39 ± 1.099	
	*I believe it is better to make important decisions even when faced with some uncertainty.*	3.51 ± 1.047	
	*When faced with uncertainty, I address the situation proactively, even if I don’t have all the information.*	3.37 ± 0.979	
	*I feel confident about maintaining employment in the future.*	3.04 ± 1.404	
	*I expect to be financially stable in the future.*	3.49 ± 1.156	
	*I believe the current economic situation influences my career decisions.*	3.54 ± 1.222	
	*I think it is important to have financial reserves because of uncertainty in the future.*	4.26 ± 0.873	
	*I am confident that I can adapt my career to future social changes.*	2.98 ± 1.107	
**Risk Preference**	*I tend to postpone making important decisions until it becomes necessary.*	2.97 ± 1.199	[12,39–42]
	*I avoid seeking opportunities because of the risks involved.*	2.51 ± 1.002	
	*I prefer to stay in my comfort zone rather than take risks.*	3.02 ± 1.172	
	*I prefer stable job options over taking chances with innovative or uncertain career paths.*	3.3 ± 1.052	
	*I avoid changing careers because I fear the unknown.*	2.73 ± 1.092	
	*I tend to wait for opportunities to come my way.*	2.78 ± 1.14	
**Adaptability & Resilience**	*I am confident in my ability to adapt to unexpected changes in my career.*	3.93 ± 0.903	[34,36,40,43–46]
	*I can handle setbacks or failures in my career without losing motivation.*	3.46 ± 1.078	
	*I am willing to retrain or upskill in order to remain competitive in the job market.*	4.17 ± 0.861	
	*I believe that resilience is a key factor in achieving long-term career success.*	3.92 ± 0.885	
	*I am open to working in different industries if my current career path faces challenges.*	3.88 ± 0.902	
	*I believe that career changes are a natural part of personal growth.*	3.73 ± 0.969	
	*I see failure as an opportunity for learning and growth.*	3.95 ± 0.991	
	*I rely on my support network (friends, family, colleagues) during difficult times.*	4.06 ± 1.001	
	*I can quickly adjust my expectations when plans do not go as I anticipated.*	3.64 ± 0.972	
**Education & Support**	*I believe that higher education is necessary for a stable future.*	3.82 ± 1.071	[36,41,46–49]
	*By completing higher education, I can secure better job opportunities and financial independence.*	3.99 ± 0.977	
	*I believe that obtaining higher or technical education is a step toward employment.*	4.17 ± 0.879	
	*I have the support of my family and friends regarding my career decisions.*	4.57 ± 0.791	

### Statistical analysis

Subsequently, collected answers and data were analysed through SPSS 29.0 software, following the approach by the authors Gwelo [52] and Hargrove, Creagh and Burgess [46]. The reliability of the questionnaire was assessed using Cronbach’s alpha, while discriminant validity was evaluated via Pearson correlations. Prior to conducting regression analysis, the underlying assumptions—linearity, independence, homoscedasticity, and normality of residuals—were tested. To address differences in scale ranges (e.g., BMLSS 0–6 vs. other constructs 1–5), all composite variables were standardized (z-scores), ensuring comparability of coefficients across predictors. The regression model was then applied to examine the impact of the constructs on career decision-making. In addition, group differences were tested using non-parametric methods: the Mann–Whitney U test for two-group comparisons (gender) and the Kruskal–Wallis test for comparisons across multiple groups (age, income, GPA, extracurricular activities engagement, desire to leave the country). These comparisons were adjusted using the Holm–Bonferroni correction method.

## Results

Internal consistency of the constructs was assessed using Cronbach’s alpha. All constructs exceeded the commonly accepted threshold of 0.70, with values ranging from 0.712 (Uncertainty) to 0.846 (Life Satisfaction). These results indicate satisfactory reliability of the measurement scales [53]. Discriminant validity was examined through inter-construct correlations. All Pearson correlation coefficients were below the 0.85 threshold, confirming discriminant validity (S1 Table) [54].

Prior to conducting regression analysis, standard assumptions were checked. Linearity and homoscedasticity were supported through visual inspection of scatterplots of standardized residuals against predicted values, which showed random distribution without clear patterns. Independence of residuals was confirmed by the Durbin–Watson statistic (1.90), indicating no problematic autocorrelation [55]. Normality of residuals was assessed through histograms, P–P plots, and Shapiro–Wilk tests, showing no significant deviations from normal distribution. Together, these results support the adequacy of the data for regression analysis.

The next step in the analysis of the survey data was the development of a regression model to further provide insights and describe the impact of composite variables on the youth career decision. The results of the regression analysis for the developed model are shown in Table 3.

**Table 3:** Summary of Regression Results.

	B	SE	β (standardized)	t	p-value	95% CI for B	Hypothesis
**Life satisfaction**	−0.042	0.031	−0.042	−1.360	0.174	[-0.102, 0.018]	H1: Rejected
**Dealing with uncertainty**	0.141	0.034	0.141	4.196	< 0.001^***^	[0.075, 0.207]	H2: Accepted
**Risk preference**	−0.222	0.034	−0.222	−6.477	< 0.001^***^	[-0.289, -0.154]	H3: Accepted
**Adaptability and resilience**	0.557	0.033	0.557	16.922	< 0.001^***^	[0.493, 0.622]	H4: Accepted
**Education and support**	0.090	0.031	0.090	2.866	0.004^**^	[0.028, 0.151]	H5: Accepted

Note. Standard multiple linear regression was applied.

*p < .05, **p < .01, ***p < .001.

R² = .462, Adjusted R² = .458

F = 114.565, p < .001

A multiple regression analysis was conducted to examine the effects of uncertainty tolerance, risk affinity, life satisfaction, adaptability/resilience, and life decisions on career decision-making. The overall model was statistically significant, F = 114.57, p < .001, explaining 46.2% of the variance in career decision-making (R² = .462, Adjusted R² = .458). VIF values were below 2, confirming the absence of multicollinearity. Results showed that dealing with uncertainty (β = .141, p < .001), education and support (β = .090, p = .004), and adaptability and resilience (β = .557, p < .001) had significant positive effects on career decision-making. In contrast, risk preference (β = –.222, p < .00) had a significant negative effect. Life satisfaction (β = –.042, p = .174) did not emerge as a significant predictor. Based on these findings, hypotheses H2, H3, H4, and H5 were supported, while hypothesis H1 was rejected.

The Mann–Whitney and Kruskal–Wallis tests further highlighted demographic and social differences among the research-defined groups in the model’s composite variables. To minimize the risk of Type I error, a Holm–Bonferroni correction was applied, as it controls the family-wise error rate while being less conservative and more powerful than the traditional Bonferroni adjustment [56]. For easier interpretation, descriptive results are presented using non-standardized data. The results are summarized in Table 4.

**Table 4:** Results of Mann Witney and Kruskal-Wallis among different groups.

		Life satisfaction	Dealing with uncertainty	Risk preference	Adaptability and resilience	Education and support	Carrer decision-making
Item	N	M ± SD	M ± SD	M ± SD	M ± SD	M ± SD	M ± SD
**Gender**							
*Male*	278	5.57 ± 0.85	3.59 ± 0.56	2.86 ± 0.73	3.84 ± 0.57	3.94 ± 0.72	4.07 ± 0.58
*Female*	395	5.60 ± 0.87	3.38 ± 0.54	2.91 ± 0.71	3.87 ± 0.54	4.27 ± 0.63	4.13 ± 0.50
***Mann Whitney p* value**		**0.556**	0.000*	**0.486**	**0.388**	0.000*	**0.535**
***Holm–Bonferroni corrected p-value***		***0.006***	0.001**	***0.005***	***0.004***	0.001**	***0.006***
**Age**							
*18-20*	594	5.54 ± 0.72	3.46 ± 0.57	2.90 ± 0.70	3.76 ± 0.62	4.12 ± 0.70	3.99 ± 0.62
*21-23*	71	5.58 ± 0.94	3.50 ± 0.55	2.86 ± 0.70	3.88 ± 0.54	4.15 ± 0.68	4.13 ± 0.51
*24-27*	8	5.61 ± 0.81	3.53 ± 0.56	2.92 ± 0.76	3.89 ± 0.53	4.13 ± 0.70	4.13 ± 0.52
***Kruskal-Wallis p* value**		***0.365***	***0.069***	0.003*	***0.624***	***0.432***	***0.903***
***Holm–Bonferroni corrected p-value***		***0.004***	***0.002***	***0.001***	***0.007***	***0.004***	***0.025***
**GPA**							
*<6.5*	118	5.54 ± 0.72	3.46 ± 0.57	2.90 ± 0.70	3.76 ± 0.62	4.12 ± 0.70	3.99 ± 0.62
*6.5-8.5*	310	5.58 ± 0.94	3.50 ± 0.55	2.86 ± 0.70	3.88 ± 0.54	4.15 ± 0.68	4.13 ± 0.51
*>8.5*	245	5.61 ± 0.81	3.53 ± 0.56	2.92 ± 0.76	3.89 ± 0.53	4.13 ± 0.70	4.13 ± 0.52
***Kruskal-Wallis p* value**		***0.507***	***0.700***	***0.804***	***0.239***	***0.972***	***0.135***
***Holm–Bonferroni corrected p-value***		***0.005***	***0.008***	***0.012***	***0.003***	***0.05***	***0.002***
**Family income**							
*500–700 €*	66	5.37 ± 1.04	3.55 ± 0.60	2.94 ± 0.69	3.88 ± 0.58	4.07 ± 0.81	4.05 ± 0.54
*700–1,000€*	91	5.23 ± 0.96	3.66 ± 0.48	2.88 ± 0.75	3.86 ± 0.63	4.18 ± 0.67	4.09 ± 0.56
*1,000–2,500 €*	242	5.57 ± 0.80	3.51 ± 0.56	2.94 ± 0.74	3.84 ± 0.51	4.22 ± 0.61	4.14 ± 0.49
*2,500–5,000 €*	229	5.76 ± 0.77	3.44 ± 0.56	2.85 ± 0.72	3.89 ± 0.57	4.09 ± 0.70	4.08 ± 0.55
*>5,000 €*	45	5.80 ± 0.80	3.42 ± 0.53	2.73 ± 0.64	3.81 ± 0.57	3.94 ± 0.83	4.14 ± 0.66
***Kruskal-Wallis p* value**		0.000*	0.013*	***0.361***	***0.816***	***0.275***	***0.708***
***Holm–Bonferroni corrected p-value***		0.001**	***0.002***	***0.002***	***0.017***	***0.003***	***0.01***
**Extracurricular activities engagement**							
*Every day*	160	5.71 ± 0.92	3.38 ± 0.58	2.72 ± 0.73	3.92 ± 0.59	4.06 ± 0.74	4.16 ± 0.58
*Several times a week*	340	5.63 ± 0.78	3.50 ± 0.55	2.86 ± 0.71	3.88 ± 0.52	4.12 ± 0.69	4.13 ± 0.50
*Weekly*	83	5.48 ± 0.74	3.53 ± 0.49	2.94 ± 0.68	3.80 ± 0.52	4.25 ± 0.62	4.02 ± 0.53
*Rarely*	83	5.34 ± 1.04	3.67 ± 0.55	3.19 ± 0.68	3.76 ± 0.60	4.24 ± 0.67	4.01 ± 0.55
*Never*	7	4.60 ± 1.10	3.72 ± 0.51	3.71 ± 0.55	3.33 ± 0.58	4.29 ± 0.53	3.75 ± 0.65
***Kruskal-Wallis p* value**		0.000*	0.000*	0.000*	0.041*	***0.169***	0.007*
***Holm–Bonferroni corrected p-value***		0.002**	0.002**	0.002**	***0.002***	***0.003***	***0.002***

GPA values are reported based on the Serbian grading scale (6–10).

*Note: p < 0.05.

**Note: Significant according to Holm-Bonferroni correction.

The Mann–Whitney U test initially indicated significant gender differences in dealing with uncertainty (p < 0.001) and education and support (p < 0.001). After applying the Holm–Bonferroni correction to control for Type I error, both differences remained statistically significant.

**Fig 3:**
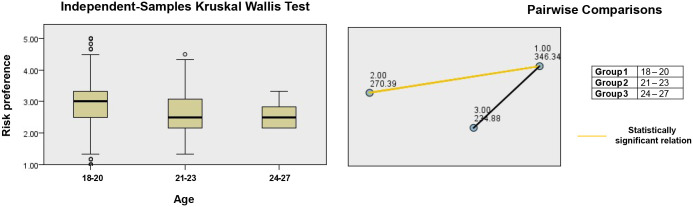
Kruskal-Wallis test scatter plot for risk preference (grouping by Age).

The Kruskal–Wallis test revealed significant age-related differences in risk preference (p = 0.003), although this effect did not remain significant after correction, and no other variables varied across age groups. The Fig 3 illustrates scatter plots and pairwise comparison chart for risk preference analysed by age group.

**Fig 4:**
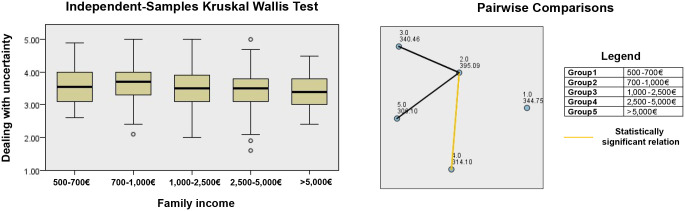
Kruskal-Wallis test scatter plots and pairwise comparisons for dealing with uncertainty (grouping by Family income).

On the other hand, regarding GPA, no significant differences were observed for any of the composite variables. In terms of family income, the Kruskal–Wallis test identified differences in dealing with uncertainty (p = 0.013), yet these disappeared following the correction. Fig 4 visually presents scatter plots and pairwise comparison chart for dealing with uncertainty analysed by family income.

**Fig 5:**
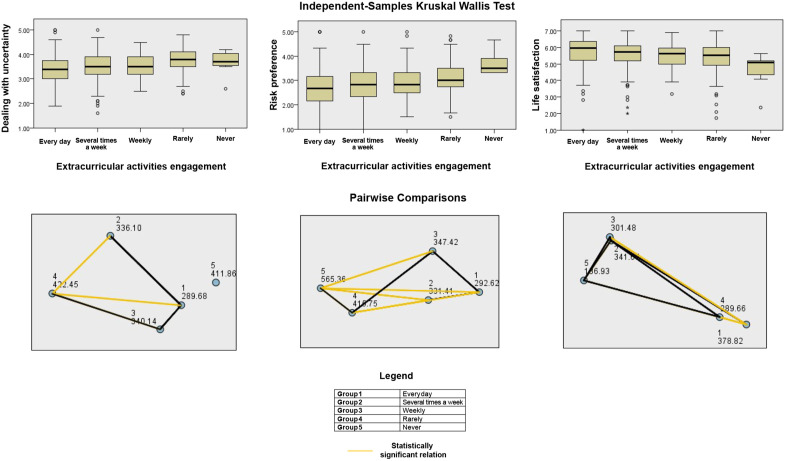
Kruskal-Wallis test scatter plots and pairwise comparisons for dealing with uncertainty, risk preference and life satisfaction (grouping by Extracurricular activities engagement).

Engagement in extracurricular activities was associated with significant differences in life satisfaction (p < 0.001), dealing with uncertainty (p < 0.001), risk preference (p < 0.001), and career decision-making (p = 0.007). After correction, the differences in life satisfaction (p = 0.002), dealing with uncertainty (p = 0.002), and risk preference (p = 0.002) remained significant, while the effect on career decision-making did not. These findings are illustrated through scatter plots and a pairwise comparison chart in Fig 5.

## Discussion

The main objective of the research was to examine the impact of key psychological and social factors on the CDM of young people, with a special focus on defined composite variables of the model, including young people deal with uncertainty, their risk preferences, education and support they receive from institutions and society, their life satisfaction and their adaptability and resilience. In this paper, the challenges of the school-to-work transition have been recognized by The European Union Youth Strategy 2019–2027 and supported by research conducted by Deloitte and UNICEF. The misalignment between support and approaches of higher education institutions and labour market demands, along with the difficulties young people face during this transition, highlight the complexity of the career decision-making process for young individuals.

Therefore, the main goal of this research was to examine the factors which influence career-decision making. According to the previously shown data analysis, it was found that the most statistically significant factors in the youth career decision model are risk preferences, dealing with uncertainty, education and support, as well as adaptability and resilience while life satisfaction was not significantly associated with career decision-making. These variables explain 46.2% of model’s variability. The remaining 53.8% of the variability may be accounted by factors not included in our model, such as career goals, readiness, orientation and other relevant elements [3,4,46,52].

### Life satisfaction

Our results indicated that life satisfaction is not significantly associated with youth career decision model, although [61] reported a significant relationship between career indecisiveness and life satisfaction. On the other hand, Yimming et al. [57] examined life satisfaction from the perspective of a mediator, where findings showed that life satisfaction does significantly mediate relationship between self-efficacy and career adaptability, but not between self-efficacy and career exploration. In line with this, our discriminant analysis showed that life satisfaction is in correlation with career adaptability, suggesting that its role may be indirect rather than direct in the career development process. Thus, the finding of our research may imply that life satisfaction does not necessarily exert a direct influence on career-decision making, but could instead operate indirectly through other variables. However, this interpretation remains tentative and requires additional empirical investigation.

Life satisfaction defers among different groups of students’ family income, as evidenced by our results, indicating that students from higher-income families reported higher level of life satisfaction. This finding underscores the importance of considering students’ socio-economic status when developing career support policies [58]. Further, this research showed that mental and physical health are important factors for life satisfaction, therefore underscoring this as another guideline and emphasizing the need for their promotion and integration as a way of enhancing the overall youth well-being. The specific importance of extracurricular activities was demonstrated by our research results, which found differences among groups with varying levels of extracurricular engagement. Hence, universities must adopt a more structured and holistic approach to embedding extracurricular activities. This can be achieved through project-based learning, accredited modules, mentorship, and partnerships, while student-led initiatives such as clubs and events further strengthen confidence and key competencies [59].

### Uncertainty

Consistent with prior studies of van den Heuvel et al. [60] our results highlight that the level of uncertainty is a critical factor affecting individual decision-making processes, with our results indicating a significantly positive association with CDM. Poor career choices with significant long-term implications can arise due to uncertainty which profoundly affects decision-making [61]. To some extent, uncertainty is unavoidable, given that a linear career development process is not a realistic expectation in the current labour market [16]. Moreover, an increase in the number of opportunities and options available to a student may lead to a rise in uncertainty [62].

This underscores the importance of providing support to students by universities, which could foster the development of uncertainty coping mechanisms, such as problem-solving and seeking social support [63], and encourage the formation of a long-term strategic perspective, serving as a foundation for key career and life decisions [64]. Career counselling and career offices have emerged as valuable solutions [65], offering students services which contribute to developing uncertainty coping mechanism, therefore enabling carefully thought-out and informed career decisions [66].

Additionally, as the results of our research showed, students engaged in extracurricular activities experience lower uncertainty levels in career decision-making, implying that universities can contribute by creating and clearly structuring certain extracurricular programs. On the other hand, our results showed that female students experience greater difficulty in coping with uncertainty, than male students. This is in line with Doruk et al [67], who notes that females tend to rely on planning and proactive approach to navigate such situations. Accordingly, universities need to offer tailored extracurricular and career support programs to address gender-specific needs, thereby equal opportunities for all students in managing career decision-related uncertainty.

### Education and support

The findings indicate that education and support are significantly positively associated with career decision-making. The identified relationship and the importance of this component are consistent with the findings of Kocak et al. [68], who reported that family and academic satisfaction influence career decision self-efficacy. This suggests that students who receive strong support from both their educational institutions and close ones tend to better manage career decision-making process compared to those who lack such support.

Providing young people with education that aligns with the contemporary needs of the labour market is of fundamental importance. Given that young people perceive education as a pathway to secure employment and stable living conditions [49], higher education institutions must continually strive to improve curricula that enhance students’ competitiveness in the labour market. Alongside formal education, support from family and friends remains a vital component [46]. In this context, it is important for educational institutions to create environments that encourage students to build and strengthen social networks, which further enhance both their career prospects and overall personal well-being [24]. Additionally, higher education institutions can offer mentoring programs that combine both academic guidance and personal support, helping students to better navigate career planning [36].

Additionally, the findings of this research showed that female students tend to place greater value on education & support more than male students. They place greater importance on social interactions, as well as on university services and support [69]. This may indicate that female students engage more actively in both the academic and social aspects of university life.

### Risk preference

The research findings highlight that risk preference is significantly negatively associated with youth career decision-making, indicating that students with lower risk preferences may navigate the career-decision making process more effectively. The identified relationship aligns with the conclusions drawn by Caner and Okten [70] who identified individual’s risk preference as one of the key determinants of career choice and the career decision-making process.

According to [12] it is important to stress that successful career decision-making results not from the extremes of either impulsive risk-taking or complete risk avoidance, but rather from a balanced risk-taking approach. The implementation of specialized training and workshops for students, aimed at enhancing their risk assessment and risk management skills could facilitate the selection and adoption of appropriate approach to career and life decision making [71]. Our findings further indicate that students differ in their risk preferences depending on their level of participation in extracurricular activities, with those more involved tending to make more risks. Therefore, students should be encouraged to take risks through different exercises, such as case studies, simulations and scenario-based exercises, as this allows universities to provide a supportive learning environment that promotes risk-taking [72], given that risk-taking is subjective and closely linked to individual’s risk affinity.

### Adaptability and resilience

Based on our findings, adaptability and resilience are significantly positively associated with career decision-making. This is in line with the general direction of findings reported by Pang et al. [73], who highlighted that resilience and career adaptability help reduce CDM difficulties. These skills are crucial for young people to meet the demands of a rapidly changing market which necessitates flexibility [7]. Additionally, our results show that career adaptability and resilience are in correlation with life satisfaction, consistent with results by Yimming et al. [57]. This underscores the value of cultivating such skills among youth, for which Verhaest & Omey [48] places emphasis on the role of formal and informal education. Providing young individuals with education and information fosters a sense of control, thereby improving motivation, preventing negative attitudes, and easing anxiety during career decision-making [74].

## Conclusions

In a world of growing uncertainty and overwhelming career choices, empowering students to make informed decisions is more important than ever. To better understand how students approach career decisions, this study identifies five key factors: Dealing with Uncertainty, Risk Preferences, Education and Support, Adaptability and Resilience, and Life Satisfaction. These constructs were used to develop a model that explains 46.2% of the variability in students’ career choices.

Four out of five factors showed statistical significance, with uncertainty, adaptability and resilience, and risk preference having the strongest impact, followed by education and support. In today’s labour market, where numerous options amplify indecision, uncertainty is a constant. Adaptability and resilience therefore may play an essential role in navigating rapid change. When it comes to risk, effective decision-making relies not on impulsiveness or avoidance, but on a balanced and thoughtful approach. Universities could consider providing adequate institutional support as well as the encouragement of social connections, which contribute to career development and decision-making. Life satisfaction was not significantly directly associated with career decision-making, but cannot be simply dismissed, as it may still be related to career decision-making process indirectly through other variables. Nevertheless, this observation requires further investigation.

This study contributes to the existing literature by offering a comprehensive model of career decision-making that includes both psychological and contextual factors. These findings suggest that universities could embed real-life decision-making exercises, such as simulations and case studies, into their curricula to help students confront uncertainty and build confidence. Career counselling services should consider expanding their scope beyond labour market information to include personalized support on life decisions such as work-life balance. Academic institutions could also begin formally recognizing extracurricular involvement and practical experience. These proposed interventions should be tested in future longitudinal or experimental studies to examine their effectiveness.

While this research provides valuable insights, several limitations should be acknowledged. The use of self-reported data collected via an online questionnaire may have introduced biases such as social desirability or inaccuracies in self-assessment. Additionally, the study relied on regression analysis, without incorporating longitudinal or qualitative methods that could offer deeper understanding of how career decision-making evolves over time. To enhance reliability, future research should apply mixed-method approaches, combining surveys with interviews or focus groups. Lastly, as the study included only students from the engineering field, the results may not be directly generalizable to students in other fields, since engineering students have specific employment opportunities and working conditions that differ from other disciplines. Furthermore, since the research was conducted only in the Republic of Serbia, the generalizability of the findings is further limited, given the country’s socio-economic characteristics. Therefore, these results may be most relevant to post-transition countries experiencing similar challenges. Expanding the research to include cross-cultural samples would provide a more globally relevant perspective.

## Supporting information

S1 Table Discriminant Validity: Inter-construct Pearson Correlations. (DOCX)

## References

[1] Lent RW, Brown SD. Career decision making, fast and slow: Toward an integrative model of intervention for sustainable career choice. Journal of Vocational Behavior. 2020;120:103448. doi: 10.1016/j.jvb.2020.103448

[2] Zhou A, Liu J, Xu C, Jobe MC. Effect of social support on career decision-making difficulties: the chain mediating roles of psychological capital and career decision-making self-efficacy. Behav Sci (Basel). 2024;14(4):318. doi: 10.3390/bs14040318 38667114 PMC11047401

[3] Kulcsár V, Dobrean A, Gati I. Challenges and difficulties in career decision making: Their causes, and their effects on the process and the decision. Journal of Vocational Behavior. 2020;116:103346. doi: 10.1016/j.jvb.2019.103346

[4] Wang X-H, Wang H-P, WenYa L. Improving the Quality of Career Decision-making of Students in Chinese Higher Vocational Colleges. Sage Open. 2023;13(2). doi: 10.1177/21582440231180105

[5] Chuang N-K, Lee PC, Kwok L. Assisting students with career decision-making difficulties: Can career decision-making self-efficacy and career decision-making profile help?. Journal of Hospitality, Leisure, Sport & Tourism Education. 2020;26:100235. doi: 10.1016/j.jhlste.2019.100235

[6] Presti AL, Capone V, Aversano A, Akkermans J. Career Competencies and Career Success: On the Roles of Employability Activities and Academic Satisfaction During the School-to-Work Transition. Journal of Career Development. 2021;49(1):107–25. doi: 10.1177/0894845321992536

[7] Taylor A. It’s for the Rest of Your Life. Youth & Society. 2005;36(4):471–503. doi: 10.1177/0044118x04268485

[8] European Union. Resolution of the Council of the European Union and the Representatives of the Governments of the Member States meeting within the Council on a framework for European cooperation in the youth field: The European Union Youth Strategy 2019–2027. Off J Eur Union. 2018 Dec 18;61(C456):1–22.

[9] Deloitte. 2024 Gen Z and Millennial survey: Living and working with purpose in a transforming world. London: Deloitte; 2024.

[10] UNICEF. Unpacking school-to-work transition: Data and evidence synthesis. (Scoping Paper No. 02). New York: UNICEF; 2019.

[11] Phillips S, Pazienza N. History and theory of the assessment of career development and decision making. In: Walsh WB, Osipow SH, editors. Career decision making. 1st ed. Hillsdale (NJ): Erlbaum; 1988. p. 1–31.

[12] Gati I, Kulcsár V. Making better career decisions: From challenges to opportunities. Journal of Vocational Behavior. 2021;126:103545. doi: 10.1016/j.jvb.2021.103545

[13] Lent RW, Brown SD, Hackett G. Social cognitive career theory. In: Brown D, editor. Career choice and development. 4th ed. San Francisco: Jossey-Bass; 2002. p. 255–311.

[14] Savickas ML. Career construction theory and practice. In: Brown SD, Lent RW, editors. Career development and counseling: Putting theory and research to work. 2nd ed. Hoboken (NJ): John Wiley & Sons; 2013. p. 144–80.

[15] Dawis RV. The Minnesota theory of work adjustment. In: Brown SD, Lent RW, editors. Career development and counseling: Putting theory and research to work. 1st ed. Hoboken (NJ): John Wiley & Sons; 2005. p. 3–23.

[16] Hartung PJ. Barrier or Benefit? Emotion in Life-Career Design. Journal of Career Assessment. 2011;19(3):296–305. doi: 10.1177/1069072710395536

[17] Di Fabio A, Palazzeschi L, Levin N, Gati I. The Role of Personality in the Career Decision-Making Difficulties of Italian Young Adults. Journal of Career Assessment. 2014;23(2):281–93. doi: 10.1177/1069072714535031

[18] Yu H, Dong Z, Guan X, Yan C, Su X, Cheng L. A Multiple Mediational Meta-Analysis of the Influence of Proactive Personality on Subjective Career Success at the Career Exploration Stage. Journal of Career Assessment. 2022;31(2):236–61. doi: 10.1177/10690727221106069

[19] Craparo G, Magnano P, Paolillo A, Costantino V. The Subjective Risk Intelligence Scale. The Development of a New Scale to Measure a New Construct. Curr Psychol. 2017;37(4):966–81. doi: 10.1007/s12144-017-9673-x

[20] Pavot W, Diener E, Colvin CR, Sandvik E. Further validation of the Satisfaction with Life Scale: evidence for the cross-method convergence of well-being measures. J Pers Assess. 1991;57(1):149–61. doi: 10.1207/s15327752jpa5701_17 1920028

[21] Serin NB, Serin O, Özbaş LF. Predicting university students’ life satisfaction by their anxiety and depression level. Procedia - Social and Behavioral Sciences. 2010;9:579–82. doi: 10.1016/j.sbspro.2010.12.200

[22] Büssing A, Fischer J, Haller A, Heusser P, Ostermann T, Matthiessen PF. Validation of the brief multidimensional life satisfaction scale in patients with chronic diseases. Eur J Med Res. 2009;14(4):171–7. doi: 10.1186/2047-783x-14-4-171 19380290 PMC3401007

[23] Burger K, Samuel R. The Role of Perceived Stress and Self-Efficacy in Young People’s Life Satisfaction: A Longitudinal Study. J Youth Adolesc. 2017;46(1):78–90. doi: 10.1007/s10964-016-0608-x 27812840

[24] An J, Zhu X, Shi Z, An J. A serial mediating effect of perceived family support on psychological well-being. BMC Public Health. 2024;24(1):940. doi: 10.1186/s12889-024-18476-z 38566105 PMC10986067

[25] Mouratidis K. Commute satisfaction, neighborhood satisfaction, and housing satisfaction as predictors of subjective well-being and indicators of urban livability. Travel Behaviour and Society. 2020;21:265–78. doi: 10.1016/j.tbs.2020.07.006

[26] Maison D. Richness: How much money do we have and how do we think about it? In: Maison D, editor. The psychology of money: An empirical study of financial behavior. Cham: Springer; 2019. p. 51–72. 10.1007/978-3-030-10570-9_2

[27] Zammitti A, Zarbo R, Magnano P, Ginevra MC. Career Adaptability, Decent Work, Meaningful Work, and Life Satisfaction in Italian Adults. The Career Development Quart. 2025;73(2):130–41. doi: 10.1002/cdq.12376

[28] Wang S-Q, Ying J, Zhang M-L, Shi Y, Li Y, Xing Z-J, et al. Health-related life satisfaction and its influencing factors: A cross-sectional study in China. Jpn J Nurs Sci. 2018;15(4):285–97. doi: 10.1111/jjns.12201 29363255

[29] Martín-Albo J, Núñez JL, Domínguez E, León J, Tomás JM. Relationships between intrinsic motivation, physical self-concept and satisfaction with life: a longitudinal study. J Sports Sci. 2012;30(4):337–47. doi: 10.1080/02640414.2011.649776 22243036

[30] Kunwijaya I, Puji Sugiharto DY, Sunawan S. Future Time Perspective and Its Influence on Life Satisfaction through Hope. JUBK. 2021;10(2):80–8. doi: 10.15294/jubk.v10i2.47990

[31] Li X, Zhang X, Lyu H. The longitudinal relationship between future time perspective and life satisfaction among Chinese adolescents. Personality and Individual Differences. 2023;202:111998. doi: 10.1016/j.paid.2022.111998

[32] Trevor-Roberts E, Parker P, Sandberg J. How uncertainty affects career behaviour: A narrative approach. Australian Journal of Management. 2018;44(1):50–69. doi: 10.1177/0312896218775801

[33] Godinic D, Obrenovic B, Khudaykulov A. Effects of Economic Uncertainty on Mental Health in the COVID-19 Pandemic Context: Social Identity Disturbance, Job Uncertainty and Psychological Well-Being Model. IJIED. 2020;6(1):61–74. doi: 10.18775/ijied.1849-7551-7020.2015.61.2005

[34] Jemini-Gashi L, Kadriu E. Exploring the Career Decision-Making Process During the COVID-19 Pandemic: Opportunities and Challenges for Young People. Sage Open. 2022;12(1). doi: 10.1177/21582440221078856

[35] Schweizer S, Lawson RP, Blakemore S-J. Uncertainty as a driver of the youth mental health crisis. Curr Opin Psychol. 2023;53:101657. doi: 10.1016/j.copsyc.2023.101657 37517166

[36] Bunjes M MS,RD, Canter DD. Mentoring: Implications for career development. Journal of the American Dietetic Association. 1988;88(6):705–7. doi: 10.1016/s0002-8223(21)02039-3 3372924

[37] Scott SG, Bruce RA. Decision-Making Style: The Development and Assessment of a New Measure. Educational and Psychological Measurement. 1995;55(5):818–31. doi: 10.1177/0013164495055005017

[38] Aven T, Renn O. On risk defined as an event where the outcome is uncertain. Journal of Risk Research. 2009;12(1):1–11. doi: 10.1080/13669870802488883

[39] Blankenstein NE, Crone EA, van den Bos W, van Duijvenvoorde ACK. Dealing With Uncertainty: Testing Risk- and Ambiguity-Attitude Across Adolescence. Dev Neuropsychol. 2016;41(1–2):77–92. doi: 10.1080/87565641.2016.1158265 27028162

[40] Yeung W-JJ, Yang Y. Labor Market Uncertainties for Youth and Young Adults: An International Perspective. The ANNALS of the American Academy of Political and Social Science. 2020;688(1):7–19. doi: 10.1177/0002716220913487

[41] Clark EC, Burnett T, Blair R, Traynor RL, Hagerman L, Dobbins M. Strategies to implement evidence-informed decision making at the organizational level: a rapid systematic review. BMC Health Serv Res. 2024;24(1):405. doi: 10.1186/s12913-024-10841-3 38561796 PMC10983660

[42] O’Higgins N. Trends in the Youth Labour Market in Developing and Transition Countries. SSRN Journal. 2003. doi: 10.2139/ssrn.758907

[43] Santilli S, Grossen S, Nota L. Career Adaptability, Resilience, and Life Satisfaction Among Italian and Belgian Middle School Students. The Career Development Quart. 2020;68(3):194–207. doi: 10.1002/cdq.12231

[44] Schloegel U, Stegmann S, Maedche A, van Dick R. Age stereotypes in agile software development – an empirical study of performance expectations. ITP. 2018;31(1):41–62. doi: 10.1108/itp-07-2015-0186

[45] Zhang Y, Camerer CF, Tashjian SM. Determinants of Economic Risk Preferences Across Adolescence. Behavioral Decision Making. 2025;38(1). doi: 10.1002/bdm.70007

[46] Hargrove BK, Creagh MG, Burgess BL. Family Interaction Patterns as Predictors of Vocational Identity and Career Decision-Making Self-Efficacy. Journal of Vocational Behavior. 2002;61(2):185–201. doi: 10.1006/jvbe.2001.1848

[47] Spencer S. Formal education and career choice. In: Gender, work and education in Britain in the 1950s. London: Palgrave Macmillan UK; 2005. p. 49–78.

[48] Verhaest D, Omey E. The relationship between formal education and skill acquisition in young workers’ first Jobs*. The Manchester School. 2012;81(4):638–59. doi: 10.1111/j.1467-9957.2012.02305.x

[49] Eriksson H, Högdin S, Isaksson A. Education and career choices: How the school can support young people to develop knowledge and decision-making skills. Univ J Educ Res. 2018;6(9).

[50] Statistical Office of the Republic of Serbia. Higher education 2023/2024. Belgrade: Statistical Office of the Republic of Serbia; 2024.

[51] Walser S, Killias M. Who should supervise students during self-report interviews? A controlled experiment on response behavior in online questionnaires. J Exp Criminol. 2011;8(1):17–28. doi: 10.1007/s11292-011-9129-5

[52] S.Gwelo A. Determinants of career choice among university students. MOJEM. 2019;7(1):1–19. doi: 10.22452/mojem/vol7no1.1

[53] Peterson RA. A meta-analysis of Cronbach’s coefficient alpha. J Consum Res. 1994;21(2):381–91.

[54] Kline RB. Principles and practice of structural equation modeling. New York: Guilford Press; 2005.

[55] Durbin J, Watson GS. Testing for serial correlation in least squares regression. II. Biometrika. 1951;38(1–2):159–78. doi: 10.2307/2332325 14848121

[56] Menyhart O, Weltz B, Győrffy B. MultipleTesting.com: A tool for life science researchers for multiple hypothesis testing correction. PLoS One. 2021;16(6):e0245824. doi: 10.1371/journal.pone.0245824 34106935 PMC8189492

[57] Yiming Y, Shi B, Kayani S, Biasutti M. Examining the relationship between self-efficacy, career development, and subjective wellbeing in physical education students. Sci Rep. 2024;14(1):8551. doi: 10.1038/s41598-024-59238-6 38609464 PMC11014916

[58] Pinto D, Sá MJ, Aguiar J, Magalhães A. Institutional policies and practices to improve access and success in higher education: the students’ proposals. European Journal of Higher Education. 2024;14(sup1):127–47. doi: 10.1080/21568235.2024.2410996

[59] Ribeiro N, Malafaia C, Neves T, Menezes I. The impact of extracurricular activities on university students’ academic success and employability. European Journal of Higher Education. 2023;14(3):389–409. doi: 10.1080/21568235.2023.2202874

[60] van den Heuvel C, Alison L, Crego J. How Uncertainty and Accountability can Derail Strategic ‘Save Life’ Decisions in Counter‐Terrorism Simulations: A Descriptive Model of Choice Deferral and Omission Bias. Behavioral Decision Making. 2010;25(2):165–87. doi: 10.1002/bdm.723

[61] Arbona C, Fan W, Phang A, Olvera N, Dios M. Intolerance of Uncertainty, Anxiety, and Career Indecision: A Mediation Model. Journal of Career Assessment. 2021;29(4):699–716. doi: 10.1177/10690727211002564

[62] Duffy RD, Blustein DL, Diemer MA, Autin KL. The Psychology of Working Theory. J Couns Psychol. 2016;63(2):127–48. doi: 10.1037/cou0000140 26937788

[63] Chaaya R, Sfeir M, Khoury SE, Malhab SB, Khoury-Malhame ME. Adaptive versus maladaptive coping strategies: insight from Lebanese young adults navigating multiple crises. BMC Public Health. 2025;25(1):1464. doi: 10.1186/s12889-025-22608-4 40259293 PMC12010593

[64] Yang Y, Zhang L, Qu W, Fan W. The effect of future self-continuity on intertemporal decision making: a mediated moderating model. Front Psychol. 2024;15:1437065. doi: 10.3389/fpsyg.2024.1437065 39176052 PMC11339553

[65] Zilai C. Empirical Research on Student Satisfaction with Career Services in Public Universities. Sage Open. 2024;14(4). doi: 10.1177/21582440241286360

[66] Wickens CD, Hollands JG. Engineering psychology and human performance. 3rd ed. Upper Saddle River (NJ): Prentice Hall. 2000.

[67] Doruk A, Dugenci M, Ersöz F, Öznur T. Intolerance of Uncertainty and Coping Mechanisms in Nonclinical Young Subjects. Noro Psikiyatr Ars. 2015;52(4):400–5. doi: 10.5152/npa.2015.8779 28360747 PMC5353115

[68] Koçak O, Ak N, Erdem SS, Sinan M, Younis MZ, Erdoğan A. The Role of Family Influence and Academic Satisfaction on Career Decision-Making Self-Efficacy and Happiness. Int J Environ Res Public Health. 2021;18(11):5919. doi: 10.3390/ijerph18115919 34072961 PMC8197847

[69] Grebennikov L, Skaines I. Gender and higher education experience: a case study. Higher Education Research & Development. 2009;28(1):71–84. doi: 10.1080/07294360802444370

[70] Caner A, Okten C. Risk and career choice: Evidence from Turkey. Economics of Education Review. 2010;29(6):1060–75. doi: 10.1016/j.econedurev.2010.05.006

[71] Zamiri M, Esmaeili A. Strategies, Methods, and Supports for Developing Skills within Learning Communities: A Systematic Review of the Literature. Administrative Sciences. 2024;14(9):231. doi: 10.3390/admsci14090231

[72] Henriksen D, Henderson M, Creely E, Carvalho AA, Cernochova M, Dash D, et al. Creativity and risk-taking in teaching and learning settings: Insights from six international narratives. International Journal of Educational Research Open. 2021;2:100024. doi: 10.1016/j.ijedro.2020.100024

[73] Pang L, Wang X, Liu F, Fang T, Chen H, Wen Y. The Relationship between College Students’ Resilience and Career Decision-Making Difficulties: The Mediating Role of Career Adaptability. PSYCH. 2021;12(06):872–86. doi: 10.4236/psych.2021.126053 PMC9145158 35624459

[74] Karabiyik T, Kao D, Magana AJ. First-Year Exploratory Studies about Students’ Career Decision Processes and the Impact of Data-Driven Decision Making. ER. 2021;5(11):418–33. doi: 10.26855/er.2021.11.003

