# Supplementary material for: School-to-work transition: The role of life satisfaction, risk perception, and resilience in youth career decision-making
Source: PLoS One. 2025 Dec 23;20(12):e0339485. doi: 10.1371/journal.pone.0339485 (PMC12725551; doi:10.1371/journal.pone.0339485)
Supplement: S1 Table — (DOCX) [file pone.0339485.s001.docx]

S1 Table. Discriminant Validity: Inter-construct Pearson Correlations.

|  | Dealing with uncertainty | Education and support | Adaptability and resilience | Risk preference | Life satisfaction | Career decisions |
| --- | --- | --- | --- | --- | --- | --- |
| Dealing with uncertainty | 1 | 0.298^**^ | 0.039 | 0.424^**^ | -0.211^**^ | 0.104^**^ |
| Education and support | 0.298^**^ | 1 | 0.258^**^ | 0.153^**^ | 0.108^**^ | 0.237^**^ |
| Adaptability and resilience | 0.039 | 0.258^**^ | 1 | -0.325^**^ | 0.274^**^ | 0.647^**^ |
| Risk preference | 0.424^**^ | 0.153^**^ | -0.325^**^ | 1 | -0.191^**^ | -0.321^**^ |
| Life satisfaction | -0.211^**^ | 0.108^**^ | 0.274^**^ | -0.191^**^ | 1 | 0.133^**^ |
| Career decisions | 0.104^**^ | 0.237^**^ | 0.647^**^ | -0.321^**^ | 0.133^**^ | 1 |

**Correlation is significant at the 0.01 level (2-tailed).
